# Casting Light on the Regulation of Platelet Reactivity by Calcitriol

**DOI:** 10.1016/j.jacbts.2026.101702

**Published:** 2026-09-22

**Authors:** Constance C.F.M.J. Baaten

**Affiliations:** a Department of Biochemistry, Maastricht University, Maastricht, The Netherlands; b Institute for Molecular Cardiovascular Research, University Hospital RWTH Aachen, Aachen, Germany; c Aachen-Maastricht Institute for CardioRenal Disease (AMICARE), Aachen, Germany

**Keywords:** calcitriol, metabolic dysfunction-associated steatotic liver disease, platelet, thrombosis

**Figure:**
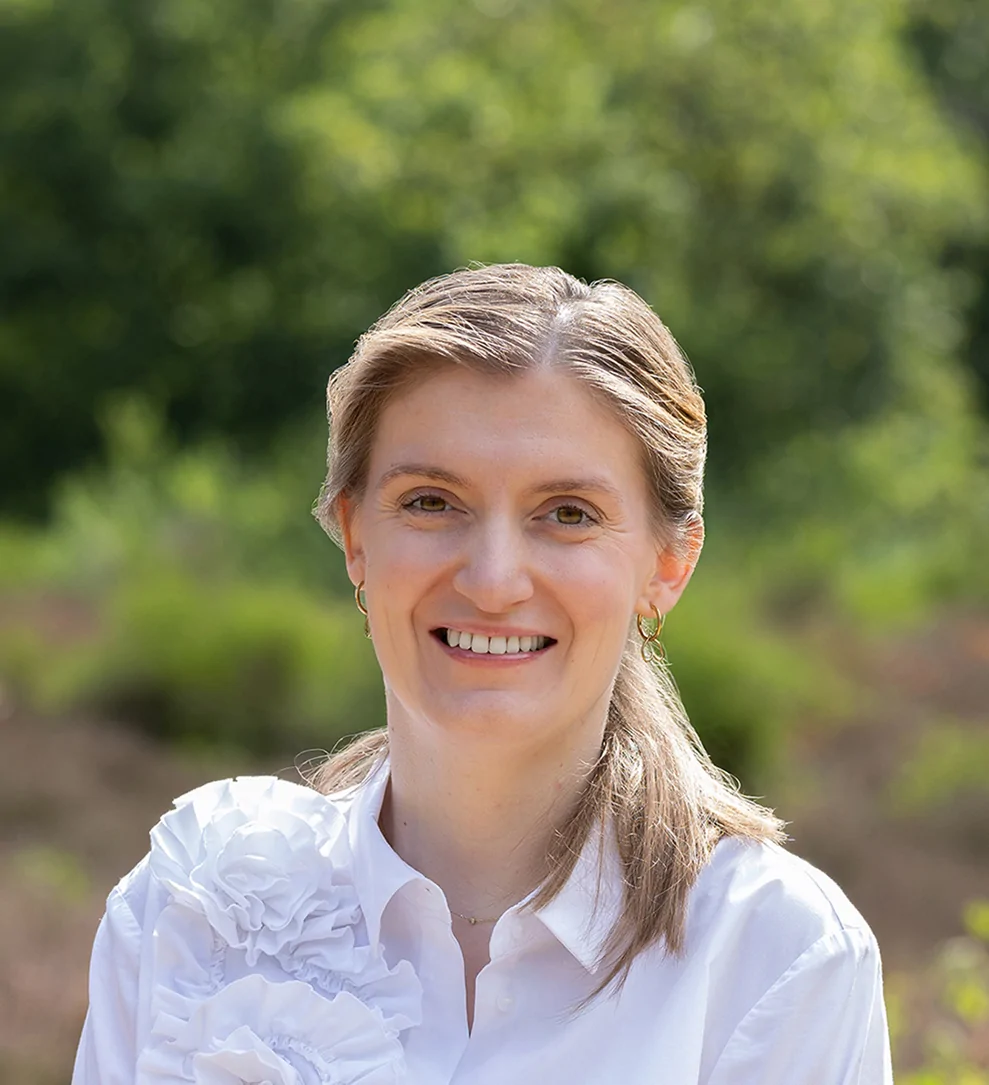

The beneficial effects of calcitriol, the active form of vitamin D, on the cardiovascular system have been the topic of many studies over the last couple of decades. Whereas observational data suggest that calcitriol provides protective cardiovascular effects because low plasma calcitriol levels are associated with an increased risk of ischemic heart disease and stroke,^1^^,^^2^ large-scale randomized clinical trials have not been able to demonstrate a reduction in cardiovascular risk upon supplementation.^3^^,^^4^ Nonetheless, multiple studies have provided evidence for a physiological role of calcitriol and its receptor, the vitamin D receptor (VDR), in the regulation of platelet activity,^5^^,^^6^ suggesting an involvement of calcitriol in the regulation of hemostasis and (athero) thrombotic events.

In the August issue of *JACC: Basic to Translational Science*, Zhang et al^7^ demonstrate a negative regulatory role of calcitriol on platelet activity in the context of metabolic dysfunction-associated steatotic liver disease (MASLD), a condition associated with a vitamin D deficiency and platelet hyperreactivity. The investigators have applied an impressive number of experimental models and setups to demonstrate the genomic and nongenomic effects by which calcitriol increases the threshold for platelet activation, both for human as well as murine platelets. At the level of the megakaryocyte, the investigators provide compelling evidence for a calcitriol-VDR-mediated down-regulation of P2Y12 expression at the mRNA as well as protein levels. The expression of other key platelet receptors such as P2Y1, proteinase-activated receptor 4, and glycoprotein VI remained unaffected. In terms of direct, nongenomic effects of calcitriol on the platelets themselves, Zhang et al^7^ showed a minor but consistent inhibition of platelet integrin activation, P-selectin expression, and platelet aggregation upon activation both in human and murine platelets after in vitro exposure to calcitriol. These findings were confirmed in samples from patients with MASLD and in mouse models of MASLD, where a vitamin D deficiency was accompanied by platelet hyperreactivity. In small-scale intervention studies, the investigators observed that supplementation of calcitriol in healthy controls subjects as well as patients with MASLD was able to elevate the plasma calcitriol levels and dampen platelet (hyper)reactivity. This reduction in platelet activity by calcitriol supplementation was also demonstrated in both control as well as MASLD mice, where calcitriol treatment led to a decrease in FeCl_3_-induced thrombosis.^7^

As shown before, platelets express the VDR.^5^ Now Zhang et al^7^ provide novel insights into platelet calcitriol—VDR signaling by drawing parallels with nongenomic VDR signaling in muscle and Sertoli cells and show that, in platelets, calcitriol also increases cyclic adenosine monophosphate levels and protein kinase A signaling in a VDR-dependent manner. This increases the threshold for platelet activation, and thereby, calcitriol enhances platelet inhibition. Additionally, incubation of platelets with calcitriol led to the VDR-dependent inhibition of the extracellular signal-regulated kinase 1/2, c-Jun N-terminal kinase, and p38 signaling pathways.^7^ Earlier work by Nie et al^6^ already showed a reduction in platelet VDR expression and an increase in the activity of the phosphoinositide 3-kinase/Akt pathway in murine platelets upon a vitamin D deficiency resulting in increased platelet activation.

The down-regulation of P2Y12 expression by calcitriol is interesting especially in light of earlier published work by Verdoia et al.^8^ They showed an association between vitamin D levels and high-on-treatment platelet reactivity (HTPR) upon adenosine diphosphate (ADP)-mediated platelet activation in patients requiring dual antiplatelet therapy for acute coronary syndrome (ACS) or after percutaneous coronary intervention (PCI) for stable coronary artery disease. Patients with a plasma vitamin D level below 9.1 ng/mL had a significantly increased platelet response to ADP compared with that in patients with adequate vitamin D levels at 30-90 days after hospital discharge. Whether this is connected to the calcitriol effects on P2Y12 expression remains to be shown. A similar association between vitamin D levels and HTPR upon arachidonic acid-mediated platelet activation could not be confirmed. However, the number of patients with arachidonic acid-mediated HTPR was low (1.2%).^8^ The association between vitamin D levels and HTPR in response to ADP was later confirmed by Verdoia et al^9^ in a cohort of patients with diabetes receiving dual antiplatelet therapy for ACS or PCI and independently by Nie et al^6^ in a cohort of patients with ACS undergoing PCI.

The observation that calcitriol supplementation led to a reduction in platelet reactivity both in healthy subjects and patients with MASLD as well as in mouse models of MASLD additionally underlines the platelet-inhibiting properties of calcitriol. It is important to emphasize though that calcitriol does not induce a complete inhibition of platelet activity. However, the effects of calcitriol are better described as a dampening of platelet activity or hyperreactivity in the context of MASLD.^7^ How these platelet effects of calcitriol connect to the lack of effect of vitamin D supplementation on the prevention of cardiovascular events as shown by earlier randomized controlled trials, remains to be understood and requires further investigation. In order to address this question, large-scale randomized controlled trials examining the effects of vitamin D supplementation in vitamin D-deficient individuals on cardiovascular outcomes should then additionally include functional measurements of platelet activity, but should preferentially also include biomarkers of coagulation, endothelial function, and inflammation as important contributors to cardiovascular outcomes. Considering the increased bleeding risk associated with current antiplatelet drugs, a strategy that dampens platelet hyperreactivity rather than inhibits platelet activity would be preferred. It remains to be shown, however, whether correcting platelet hyperreactivity by vitamin D supplementation is sufficient to prevent the development of atherothrombotic events.

## Funding Support and Author Disclosures

Dr Baaten has reported that she has no relationships relevant to the contents of this paper to disclose.
